# Clinical outcomes of the PAUL® Glaucoma implant for refractory glaucoma: three-year results

**DOI:** 10.1038/s41433-025-04131-3

**Published:** 2025-12-16

**Authors:** Constance Liegl, Leonie Bourauel, Benjamin Aretz, Wolfgang Walz, Sarah Hundertmark, Michael Petrak, Frank G. Holz, Karl Mercieca

**Affiliations:** 1 Department of Ophthalmology, University of Bonn, Bonn, Germany; 2 Institute for Medical Biometry, Informatics and Epidemiology, University of Bonn, Bonn, Germany

**Keywords:** Outcomes research, Predictive markers

## Abstract

**Purpose:**

To report three-year outcomes undergoing PAUL® Glaucoma Implant (PGI) surgery in a single-centre cohort.

**Methods:**

A retrospective cohort study including patients undergoing PGI surgery at the University Eye Hospital Bonn, Germany, from April to September 2021. Patients were enrolled in a database at the time of surgery, with follow-up data collected at each visit. The primary outcome was the success rate based on predefined IOP criteria (A ≤ 21, B ≤ 18, C ≤ 15, and D ≤ 12 mmHg). Secondary outcomes included IOP, BCVA, IOP-lowering medications, complications, and intraluminal Prolene stent removal.

**Results:**

Sixty eyes of 56 patients were included. Qualified and complete success rates (95% CI) were 88% (80–97) and 43% (30–56) for Criterion A, 67% (53–78) and 37% (23–50) for Criterion B, 43% (30–57) and 27% (15–39%) for Criterion C and 30% (18–42) and 22% (12–33) for Criterion D. Mean IOP decreased from 26.73 mmHg (7–48) to 10.83 mmHg (4–20) (reduction of 54%) over 36 months with a reduction in IOP-lowering agents from 3.38 to 0.57. Three eyes developed hypotony requiring intervention, one required DMEK for corneal decompensation, and seven required conjunctival revision for tube exposure. The Prolene stent was removed in 29 eyes (49.2%) after a mean of 4.44 months, reducing IOP from 23.58 to 12.04 mmHg.

**Conclusions:**

PGI surgery effectively reduces IOP and medication burden in refractory glaucoma, with sustained efficacy over three years. The intraluminal Prolene stent enables further non-invasive IOP reduction postoperatively.

## Introduction

Glaucoma drainage devices (GDD) are often used for treating eyes with secondary glaucoma, such as cases of uveitic or neovascular glaucoma. They can also be used to surgically manage chronic primary open-angle glaucoma (COAG), particularly when initial filtering glaucoma surgery has failed or when there is conjunctival scarring from previous ocular procedures [1–3]. The *Ahmed versus Baerveldt* study (AVB) [4]and the *Ahmed Baerveldt Comparison* study *(ABC)* [5]compared the Ahmed Valve Glaucoma Implant (AGI) with the Baerveldt Glaucoma Implant (BGI) with both studies finding that the valveless BGI achieved a greater long-term reduction in intraocular pressure (IOP). However, the BGI was associated with a higher incidence of postoperative hypotony [6]. The PAUL® Glaucoma Implant (PGI) (Advanced Ophthalmic Innovations, Singapore) was designed with a plate size and valveless tube similar to the BGI but it features smaller tube diameters both internally (0.127 mm) and externally (0.467 mm). Early outcomes of the PGI indicate that it effectively lowers IOP and reduces the need for glaucoma medications, while maintaining a low complication rate, particularly in terms of postoperative hypotony. So far, only two studies have provided three-year data demonstrating good effectiveness and safety. Tan et al. published three-year results from Singapore, focusing on an Asian population [7]. Richardson et al. presented three-year data exclusively on uveitic patients [8]. Other studies with one- or two-year data published to date have also demonstrated positive results [9–12].

In this study, we report the results and outcomes of the very first cases of PGI in Germany, primarily involving a Caucasian population, which include 60 eyes of 56 consecutive patients with a follow-up of at least three years. We also describe our experience with an intraluminal stenting technique to avoid early postoperative hypotony, as described by previous studies [9, 12].

## Materials/subjects and methods

### Patients

The medical records of all patients who underwent PGI surgery at the Department of Ophthalmology at the University Hospital of Bonn, Germany, from 04/2021 to 09/2021 were reviewed. Patients were enrolled in a database at the time of surgery, and follow-up data were prospectively collected during each postoperative visit. All patients underwent a comprehensive ophthalmic examination upon presentation, which included the assessment of best-corrected visual acuity (BCVA) using the Snellen chart (converted to logMAR for statistical analysis), IOP measurement via Goldmann applanation tonometry, slit lamp biomicroscopy, fundus biomicroscopy and visual field testing, using the Humphrey 24-2 (Carl Zeiss Meditec, Inc., Dublin, CA) visual field test strategy. For each consecutive patient who received a PGI implant, the preoperative data collected included gender, age, type of glaucoma, BCVA, clinical features (e.g., IOP and anterior segment signs), and detailed follow-up data on BCVA, IOP, visual fields, complications, and postoperative glaucoma medications. Four success criteria (based on WGA guidelines) were applied, with IOP thresholds as follows:

1. Criterion A: IOP ≤ 21 mmHg,
2. Criterion B: IOP ≤ 18 mmHg,
3. Criterion C: IOP ≤ 15 mmHg, and
4. Criterion D: IOP ≤ 12 mmHg.

Success was classified as complete/cumulative if achieved without glaucoma medications and as qualified if achieved with medication and was assessed at each postoperative time point, beginning three months after surgery. PGI surgery was considered a failure in cases of persistent IOP below 6 mmHg after three months post-surgery, the need for additional glaucoma surgery for uncontrolled IOP, PGI explantation, or loss of light perception. If IOP was above thresholds before prolene removal, the IOP after prolene removal was counted in order to determine whether the respective threshold was reached.

The primary outcome was the success rate based on the defined criteria. Secondary outcomes included IOP, BCVA, the number of IOP-lowering medications, complications, and details regarding polypropylene intraluminal stent removal. All analyses were performed on a de-identified dataset, and the study was approved by the Ethics Committee at the University Hospital Bonn (number: 458/21). The protocol adhered to the ethical standards of the 2000 Declaration of Helsinki, as confirmed by the institution’s Human Research Committee.

### Statistical analysis

Statistical analysis was performed with SPSS Statistics version 27.0.0 (IBM Corporation, New York) and R 4.4.2 (R Core Team 2021) [13]. The paired sample t-test was used to test differences between each pair of time points. To predict IOP at follow-up, we employed a Generalised Additive Model (GAM) to allow for nonlinear effects, particularly of preoperative IOP. A thin plate regression spline was used to flexibly model this association. The GAM assumed a Gamma distribution with a log link to account for the skewed nature of the outcome.

Model parameters were estimated via Restricted Maximum Likelihood (REML), and residual diagnostics indicated an adequate fit.

#### Surgical approach

All surgeries were performed by the same experienced, fellowship-trained glaucoma surgeon (KM). The PGI is usually placed in the superotemporal quadrant but may be alternatively positioned superonasally or inferonasally when necessary. The superotemporal quadrant is regularly chosen since this location allows plate and bleb cover by the upper lid and also offers good surgical access. Conjunctival and Tenon’s peritomy result in exposure of the superotemporal quadrant with a fornix-based flap. Mitomycin-C (MMC) at a concentration of 0.5 mg/ml is applied to the location of the PGI plate for two minutes. The plate is then placed under the recti muscles and sutured to the sclera with 9.0 nylon sutures, 10 mm away from the corneal limbus. A 6.0 polypropylene thread is inserted into the PGI tube. A 26 G cannula is used to create a tunnel into the anterior chamber. The tube is shortened to the required length, placed through the tunnel and secured with a nylon 9/0 ‘box’ suture. A double-layered pericardial patch graft is used to cover the tube (fibrin glue used for adhesion) and the prolene thread is usually placed in an inferior sub-conjunctival pocket. The conjunctiva is closed with two 10/0 nylon corner slip knots and two 10/0 nylon mattress sutures.

The polypropylene stent is removed during the postoperative period when the IOP rises above target level. While there was no fixed timeframe for removal, it is performed as needed, but usually never within the first eight weeks after surgery.

## Results

60 eyes of 56 consecutive patients who underwent PGI surgery were included in this study. Nearly all patients were of Caucasian ethnicity (55 pat., 99.2%); only one patient was Southeast Asian (1.8%). The mean age was 63.2 years (14-85 y). Most eyes had COAG (27 eyes, 45%) or secondary glaucoma (18 eyes, 30%). 37 eyes (61.7%) had undergone previous glaucoma surgery, and 10 eyes (16.7%) had previously had a pars plana vitrectomy. All PGI were placed in the superotemporal quadrant. Other patient characteristics are shown in Table 1.

**Table 1:** Demographics and clinical characteristics of patients undergoing PGI surgery.

Demographics and clinical characteristics of eyes undergoing PGI surgery	
	n = 60 eyes, 57 patients
**Gender**	
Male / female	29 (48.3) / 31 (51.7)
**Ethnicity**	
Caucasian	56 (98.2)
Southeast Asian	1 (1.8)
**Age**	
Mean (Range)	63.2 (14–85)
**Glaucoma type**	
POAG	27 (45)
PEX glaucoma	9 (15)
Secondary glaucoma	18 (30)
PACG	1 (1.7)
Others	5 (8.3)
**Previous glaucoma surgery**	
Yes	37 (61.7)
**Which surgery**	
Trabeculectomy	12 (20)
XEN	6 (10)
Canaloplasty	4 (6.7)
Trabectome	4 (6.7)
CPC	24 (40)
Preserflo Microshunt	1 (1.7)
Deep Sclerectomy	1 (1.7)
iStent	2 (3.4)
**Anaesthesia**	
Local / General	20 (33.3) / 40 (66.7)
**Number (#) of glaucoma drops**	
Mean (Range)	3.38 (1–5)
**Acetazolamide**	
Yes	16 (26.7)
**Lens status**	
Phakic. Pseudophakic;Aphakic	18 (30) / 41 (68.3) / 1 (1.7)
**BCVA preoperatively (logMAR)**	
Mean (Range)	0.72 (0–2.7)
**IOP preoperatively**	
Mean (Range)	26.73 (7–48)
**Maximum IOP preoperatively**	
Mean	35.12 (17–79)
**Mean Deviation (MD) (Humphrey 24-2)**	
Mean	−14.62 (−0.47–−35.26)
**Pattern Standard Deviation (PSD)**	
Mean	6.54 (1.68–13.33)

The mean preoperative IOP was 26.73 (7-48 mmHg). All patients were using topical IOP-lowering therapy with a mean of 3.38 agents (1–5 agents).

**Fig. 1:**
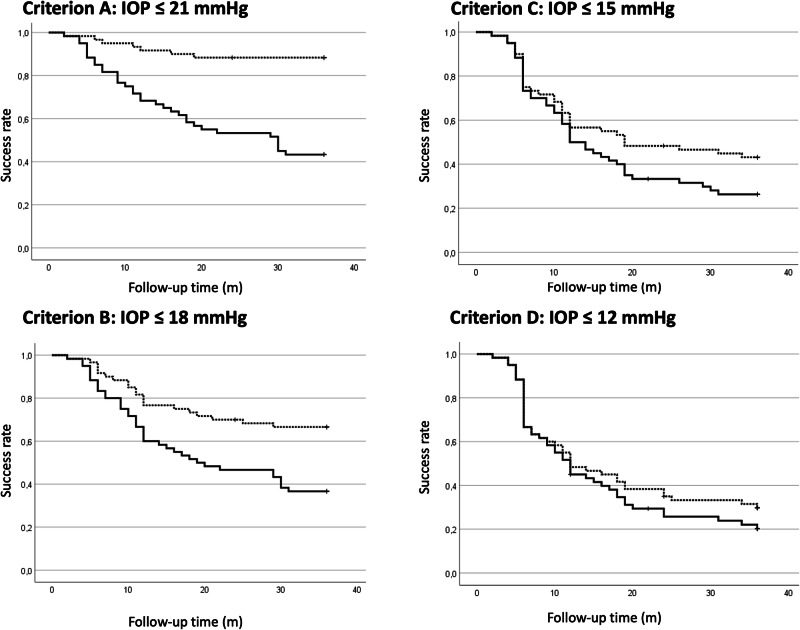
Kaplan–Meier curves. Complete and qualified success rates (95% CI) were 51.8% (37.5–66.1) and 89.3% (80.4–96.4) for Criterion A, 48.2% (35.7–60.7) and 78.6% (67.9–87.5) for Criterion B, 44.6% (32.1–57.1) and 64.3% (51.8–76.8%) for Criterion C and 26.8% (16.1–39.3) and 37.5% (25.0–50.0) for Criterion D.

Primary outcomes were related to the above-mentioned success criteria. Qualified and complete success rates (95% CI) were 88% (80–97) and 43% (30–56) Criterion A (IOP ≤ 21 mmHg), 67% (53–78) and 37% (23–50) for Criterion B (IOP ≤ 18 mmHg), 43% (30–57) and 27% (15-39%) for Criterion C (IOP ≤ 15 mmHg) and 30% (18–42) and 22% (12–33) for Criterion D (IOP ≤ 12 mmHg) (Fig. 1).

Five eyes (8.3%) developed a failure event due to explantation of the PGI following persistent tube exposure despite surgical revision with a second patch graft, one eye received a cyclophotocoagulation (CPC) during the postoperative course (1.7%).

**Fig. 2:**
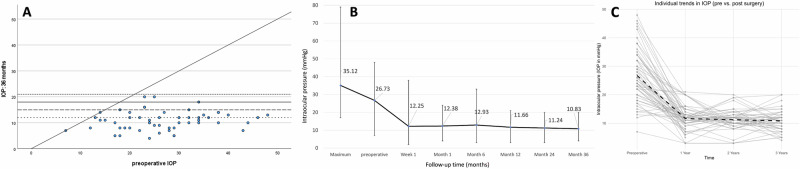
Graphical Illustration of IOP development. **A** Scatterplot of preoperative versus postoperative IOP values at 3 years after surgery. Dots represent eyes, horizontal lines indicate IOP used as threshold for success criteria,** B** IOP development: Mean IOP decreased from 26.73 (7–48 mmHg) to 10.83 mmHg (4–20 mmHg) with a mean reduction of 54.69% (0–83.7%) respectively after 36 months. **C** Individual intraocular pressure (IOP) trajectories over time following PGI implantation. Each line represents one patient’s IOP measured preoperatively and at 1, 2, and 3 years postoperatively. The plot illustrates the overall trend of decreasing IOP following surgery, with most eyes maintaining sustained IOP reduction through year 3. Minor fluctuations reflect individual variability in response and long-term control.

Following PGI implantation in our cohort, the mean IOP decreased from 26.73 (7-48 mmHg) to 9.57 (3–22 mmHg) on day 1 with a mean reduction of 60.33% (0–89.7%); 11.66 mmHg (3–21 mmHg) with a mean reduction of 50.94% (0–90.6%) at 12 months, 11.24 mmHg (3–20 mmHg) with a mean reduction of 52.24% (0–89.7%) after 24 months and 10.83 mmHg (4–20 mmHg) with a mean reduction of 54.69% (0–83.7%) respectively (Fig. 2A–C). IOP values for other time points can be found in Table 2.

**Table 2:** IOP, percentage reduction, BCVA and pressure-lowering eye drops during the postoperative course.

IOP and percentage reduction during the postoperative course			
Time point	n	IOP (range)	Percentage reduction from preoperative IOP
***Maximum IOP***	60	35.12 (17–79)	n/a
***Preoperative***	60	26.73 (7–48)	n/a
***Day 1***	60	9.57 (3–22)	60.33 (0–89.7)
***Month 1***	60	12.38 (4–24)	51.33 (8.7 - 81.8)
***Month 3***	56	12.46 (4–22)	48.67 (0–80.0)
***Month 6***	54	12.93 (3–33)	47.31 (5.7–84.4)
***Month 12***	56	11.66 (3–21)	50.94 (0–90.6)
***Month 18***	53	11.44 (3–20)	53.08 (0–100)
***Month 24***	49	11.24 (3–20)	52.24 (0–89.7)
***Month 30***	51	10.46 (4–20)	53.92 (0–83.7)
***Month 36***	54^a^	10.83 (4–20)	54.69 (0–83.7)
**BCVA and pressure-lowering eye drops**			
		**BCVA**	**# of agents**
***Preoperative***	60	0.72	3.38
***Month 1***	60	0.76	0.33
***Month 6***	54	0.76	0.33
***Month 12***	56	0.83	0.41
***Month 24***	49	0.75	0.45
***Month 36***	54^a^	0.79	0.57

^a^6 eyes were censored from further follow-up due to failure and further glaucoma surgery.

The mean number of IOP-lowering agents was reduced from 3.38 (1–5) to 0.41 (0-3), 0.45 (0–3) and 0.57 (0–3) at 12, 24 and 36 months respectively. Whilst 16 eyes (26.7%) needed systemic acetazolamide therapy preoperatively, no patient required it at any time point after surgery.

Median BCVA was 0.6 logMAR (0–2.7) preoperatively and decreased after surgery to 0.8 logMAR (0.1–2.7) on day 1 and week 1, respectively. However, the BCVA slightly deteriorated in comparison to preoperative levels by 36 months after surgery with a median BCVA of 0.8 logMAR (0–2.7). This visual deterioration was caused by ocular comorbidities. Two eyes (3.4%) developed retinal detachment and were treated with pars plana vitrectomy. One eye (1.7%) developed a retinal vein occlusion and needed intravitreal injections.

Postoperative complications were observed in 16 eyes (26.7%). Three eyes (5%) had an intracameral injection of viscoelastic due to significant hypotony with AC shallowing, with one of these eventually needing external tube ligation due to persistent low IOP. Two eyes (3.4%) developed aqueous misdirection which resolved after a YAG-laser hyaloidotomy in one eye and after vitrectomy in the other eye. Two eyes (3.4%) had a permanent corneal decompensation and one of these received a Descemet membrane endothelial keratoplasty (DMEK) and due to failure a penetrating keratoplasty. Seven eyes (11.7%) developed a tube exposure which required conjunctival revision with additional pericardial patch graft, of these five eyes underwent a PGI explantation despite multiple revisions. Further complications are illustrated in Table 3. Six eyes (10%) needed additional glaucoma procedures that led to failure of the surgery (Table 3).

**Table 3:** Complications and failure events following PGI surgery.

Time Point	Complications	Number of pat. (%)
**Complications overall**		16 (26.7)
Intraoperative	Corneoscleral defect after previous trab and patchgraft	1 (1.7)
First week postoperative	Hyphaema	3 (5.0)
	Hypotony with choroidal detachment -Injection of viscoelastic -External tube ligature	5 (8.3) −3 (5.0) −1 (1.7)
First three months postoperative	Postoperative macular oedema and peribulbar corticosteroid injection	3 (5.0)
	Aqueous misdirection -Resolved by YAG-Iridozonulohyaloidotomy -Resolved after vitrectomy	2 (3.4) −1 (1.7) −1 (1.7)
	Double vision	1 (1.7)
After three months postoperative	Hypotony after prolene removal and external ligature	1 (1.7)
	Corneal decompensation -DMEK -Same eye: Keratoplasty after failed DMEK	2 (3.4) −1 (1.7) −1 (1.7)
	Revision for tube erosion	7 (11.7)
	Reposition of tube due to anterior position and endothelial cell loss	1 (1.7)
**Additional glaucoma surgeries that lead to failure after PGI**		
CPC		1 (1.7)
PGI-explantation and re-implantation in other quadrant		2 (3.4)
PGI explantation		1 (1.7)
PGI explantation and Preserflo Microshunt implantation		1 (1.7)
PGI explantation and AGI implantation		1 (1.7)

An intraluminal stent in the form of a 6-0 polypropylene thread was used in every case of this cohort. This was removed in 27 eyes (45%) after a mean time period of 4.44 months (1–26 months). Mean IOP before removal was 23.58 mmHg (12–40) and decreased to 12.04 mmHg (6–22). There were no complications related to its removal.

A regression analysis was conducted to identify factors associated with postoperative IOP. No significant correlations were found for glaucoma type (POAG vs. PEX glaucoma with: β = 0.102, p = 0.520, angle closure glaucoma with: β = -0.089, p = 0.814, secondary glaucoma with: β = 0.026, p = 0.855, others with: β = 0.118, p = 0.605), age in quantiles (2.quantile: β = -0.099, p = 0.535, 3. quantile: β = 0.115, p = 0.189, 4. quantile: β = -0.247, p = 0.226), gender (females vs. males with: β = 0.023, p = 0.831), number of pressure-lowering eye drops, use of acetazolamide (no vs. yes with: β = -0.062, p = 0.649), lens status (phakic/ pseudophakic vs. aphakic with β = -0.109, p = 0445), or previous glaucoma surgeries (no vs. yes with: β = -0.058, p = 0.695). Besides preoperative IOP (β = 0.099, p < 0.001), the only factor that demonstrated a significant association with IOP was visual acuity (normal vision logMAR <0.3 (reference), mild impairment: logMAR 0.3–0.5 (β = 0.060, p = 0.642); moderate impairment: logMAR 0.6–1.0 (β =-0.971, p = 0.015): severe impairment: logMAR 1.1–1.3 (β = 0.109, p = 0.632); (almost) blindness: logMAR ≥ 2.0 (β =-0.060, p = 0.787)). Patients with moderate preoperative visual acuity experienced a greater IOP-lowering effect compared to patients with normal vision.

## Discussion

This study highlights the efficacy of the PGI at three years, with qualified and complete success rates of 88% and 43% for achieving an IOP of 21 mmHg or less. To date, only one other study, which included 48 eyes, has published three-year PGI outcomes for all glaucoma types [7] while another published data solely on uveitic eyes (50 eyes). With 60 eyes, our study therefore represents the largest, generalised, 3-year outcomes PGI cohort so far. Comparing success rates is challenging due to differing criteria. Tan et al. required IOP > 18 mmHg or < 6 mmHg on two consecutive visits to define failure, aligning with our success criterion B (IOP ≤ 18 mmHg), though we considered success achieved if the threshold was exceeded at any single time point. As in our study, they defined complete success as „the absence of failure without the use of IOP-lowering medications“. Consequently, their failure rate (14.6%) and our success rates (67% and 37%) for IOP ≤ 18 mmHg are not directly comparable. For a secondary criterion at 15 mmHg, comparable to our criterion C, Tan et al. reported an 85.4% success rate (75% complete), while ours were lower at 43% and 27%. This discrepancy likely reflects the difference in requiring two consecutive high IOP readings versus a single elevated value. Richardson et al. defined success as IOP ≤ 21 mmHg and ≥ 5 mmHg with a ≥ 20% IOP reduction, reporting 48% complete and 92% qualified success rates. However, their study is not directly comparable as it exclusively included patients with uveitic glaucoma.

In order to compare clinical outcomes further, we looked at mean IOP as well as the number of IOP-lowering eye drops. Previous data from our two-year results and previous studies had shown an effective IOP reduction after two years [9–11, 14].

Regarding three-year results, Tan et al. reported a preoperative IOP of 20.6 mmHg (+/− 6.13 mmHg) with a reduction to 14.9 mmHg (+/− 4.11 mmHg) after 36 months. The preoperative IOP in our study was higher at 26.73 (7-48 mmHg) and was reduced to 10.83 mmHg (4–20 mmHg) after three years, thus showing a greater overall IOP reduction and lower final mean IOP. Tan et al. had a mean number of IOP-lowering drops of 3.13 ( + /− 0.959) which decreased to 0.167 ( + /− 0.476) after three years. Our study had a similar proportion of drops pre-operatively which reduced from 3.38 (1–5) to 0.57 (0–3) at 36 months. The mean number of postoperative IOP-lowering medications in our study was higher, likely due in part to increased caution in removing the polypropylene stent, particularly with IOPs in the higher teens. Additionally, the use of an intraluminal stent may contribute to slightly elevated IOP or a greater reliance on medications. One of our patients developed persistent hypotony during the postoperative course after polypropylene removal, requiring an external ligature. This highlights the risk of hypotony when the stent suture is removed prematurely. In these patients we now tend to commence topical aqueous suppression to avoid the risk of a dramatic IOP drop and we keep polypropylene removal as a potential future option for lowering IOP if it becomes necessary. Richardson et al. showed a reduction of IOP from 30.6 mmHg to 12.2 mmHg with a decrease of medication from 3.9 (and 62% with acetazolamide) to 1.1. These results are more similar to ours. Comparison with this study is challenging, as it exclusively included uveitic eyes. However, since the same (polypropylene) technique was used, the similarity in results is reasonable. The group of Tan et al. did not use an intraluminal stent, but a different approach to prevent hypotony. In place of a stent, they used a pericardial patch graft inserted between the subconjunctival space and PGI plate with cross-linked viscoelastic injected beneath and above the patch.

Further, in comparison to Tan et al. who did not use Mitomycin C (MMC) as a antifibrotic agent, we used MMC in all eyes of our study except for two eyes of a pregnant woman. Antimetabolites such as mitomycin C (MMC) are often used in GDI surgery to prevent scarring, but most of the current studies do not see a clear advantage in the regular use of MMC [15, 16]. Even though there is no clear evidence that the usage of MMC leads to better results, it might have attributed to the fact that the final IOP in our study was slightly lower in comparison to Tan et al.

Six eyes (10.2%) in our study underwent additional glaucoma procedures and / or PGI explantation leading to failure and exclusion from follow-up, five of which were due to a persistent exposure with need for explantation. The latter were not taken into account for IOP values after 36 months, so that in total we only included the follow-up data of 54 eyes. This might also have attributed to the lower final IOP values in comparison to Tan et al. In their study, no eye needed a tube explantation and none received additional glaucoma surgery, so that they included the IOP of their whole cohort after 36 months, including more “problematic” eyes. This might have contributed to their higher postoperative IOP values.

In view of the paucity of 3 year data for PGI, it is important to compare the currently available data to the landmark studies involving the AGV and BGI with the same three-year time frame. When looking at the Ahmed-Baerveldt comparison study (ABC) [5]and Ahmed versus Baerveldt Study (AVB) [4], the IOP was 14.3 mmHg (ABC) and 15.7 mmHg (AVB) for the AGV and 13.1 mmHg (ABC) and 14.4. mmHg (AVB) for the BGI, respectively. The number of IOP-lowering agents was 2.0 (ABC) and 1.8 (AVB) for the AGV and 1.5 (ABC) and 1.1 (AVB) for the BGI. Thus, our study showed a lower final IOP for the PGI after three years with a smaller number of pressure-lowering agents. Tan et al. also reported on a lower amount of medication whilst their IOP was rather similar. All in all, these results seem promising and underline that the PGI was still functioning well after three years maintaining the positive IOP-lowering effect.

Apart from the success rates and pressure-lowering effect of new devices, it is important to look at safety aspects and complication rates. The ABC and AVB concluded that the valveless BGI led to higher hypotony and complication rates whilst the AGV was considered safer regarding this aspect. However, the BGI resulted in lower IOP values. The PGI offers the advantage of a smaller tube lumen whilst also being valveless, so that in theory the risk of hypotony should be lower whilst the more powerful IOP-lowering effect of a valveless implant can be achieved. In our study, we included clinically significant hypotony with choroidal detachment and/or macular folds and did not include simple numerical hypotony values. A total of 5 eyes (8.3%) developed hypotony with choroidal detachment. Of these, 2 eyes (3.4%) required viscoelastic injection for AC reformation, and 1 eye (1.7%) needed an additional external tube ligature, despite the use of an intraluminal polypropylene stent. Tan et al. reported much higher hypotony rates (35.4%) but this included numerical hypotony. Of these, 4 eyes required an AC reformation. Further, they had four eyes of shallow anterior chambers not associated with hypotony with two of these needing an AC reformation as well, presumably due to aqueous misdirection. In our cohort, we had two cases of the latter. This might be due to our use of an intraluminal polypropylene stent for partial occlusion of the tube in order to prevent early hypotony and enable further IOP lowering at a later date by removal at the slit-lamp. As described before, the group of Tan et al. used a different approach to prevent hypotony utilising viscoelastic beneath and above the patch. They concluded that this method obviates the need for postoperative stent removal whilst it may have been contributed though to the variability of hypotony in their cohort. Further, as shown in this study, our final IOP values were lower than by Tan et al. which might have also been due to the possibility of removing an intraluminal polypropylene stent leading to a further IOP reduction during the postoperative course [7]. Richardson et al. also used an intraluminal polypropylene stent that was removed in 42% of eyes leading to a reduction from 24.7 mmHg to 14.3 mmHg without any associated cases of hypotony. Their overall hypotony rate was 10% and thus, also lower than in the study of Tan et al.

In our cohort, seven eyes (11.7%) required revision surgery due to tube exposure, a rate which is high compared to other PGI studies, such as Tan et al. who reported no such events. All eyes in our cohort had undergone previous ocular surgeries—including trabeculectomy or CPC—and represented complex, high-risk cases. However, this is expected in tube surgery patients and was not particularly different from other studies. All implants were placed in the superotemporal quadrant, and surgeries were deemed uncomplicated. While MMC likely contributes to effective IOP control, anterior application—if inadvertently extending beyond the plate area—may play a role in patch graft thinning and exposure. In all our patients, MMC was applied at a concentration of 0.5 mg/mL for 2 min, taking meticulous care to confine application to the plate area. Tube exposure was anterior in all cases, rather than over the plate, which goes against MMC being a causative factor in our cohort. Conjunctival handling or graft material may therefore have been factors. Our conjunctival closure technique routinely involves the use of two slip-knot corner sutures and two mattress suture across the limbus with fibrin glue application used for relaxing incisions. While this approach generally provides good coverage, it may not have prevented conjunctival retraction in all cases. Bovine pericardium was used as patch material in these cases and, despite the fact it was double-layered, showed complete dissolution within the first few months of surgery. Other centres have reported lower erosion rates with double-layered pericardium, including our experienced surgeon (KM) in this cohort who previously worked in and published PGI data from a different eye clinic [8]. The authors strongly feel that the increased exposure rate and, as a consequence, higher rates of explantation were related to the pericardial material used in our first year of PGI in our centre. A switch to fascia lata in the second year has resulted in virtually no tube erosions at all over a 3 year period [17].

Two cases of retinal detachment occurred in our cohort following PGI implantation. In one patient, the detachment represented a re-detachment in an eye with a history of vitreoretinal surgery for rhegmatogenous retinal detachment, complicated by secondary glaucoma. Given the pre-existing retinal pathology and surgical history, the re-detachment was likely attributable to the underlying disease rather than the PGI procedure itself. The second case occurred in a patient who had previously undergone pars plana vitrectomy for an epiretinal membrane. While a direct causal link to the PGI cannot be established, the prior vitreoretinal intervention may have predisposed the eye to retinal instability, contributing to the development of the detachment postoperatively.

This study has several limitations, including its retrospective nature. Although data was prospectively collected after every consecutive patient visit, we did not schedule study-specific appointments with some resulting loss to follow-up. Despite even reaching out telephonically to both patients and local ophthalmologists, we still did not manage to gather three-year data for every consecutive patient operated during the first months after PGI introduction. This inevitably introduces a selection bias. A prospective randomised clinical trial in the future would be beneficial to obtain compare different techniques. This study was also non-comparative and thus does not give information on how the PGI compares with other established GDD. Further, different techniques in order to prevent hypotony could be compared that way. We also chose IOP control as one of our main outcome measures. This surrogate is an imperfect value for structural and functional success. Another limitation is the inclusion of different types of glaucoma, making it difficult to generalise these results to a broader patient population.

## Conclusion

Our results show that the PGI is a safe and effective GDD which provides effective IOP lowering with a low complication rate, especially with regards to postoperative hypotony, even after three years. ´The use of an intraluminal 6/0 polypropylene stent helps reduce the rate of postoperative hypotony whilst also allowing immediate aqueous outflow and its removal leads to further IOP reduction during the postoperative course.

## Summary

### What was known before

- The PAUL® Glaucoma Implant (PGI) effectively lowers intraocular pressure (IOP) and reduces the need for pressure-lowering medications for up to one to two years.
- PGI has shown promising success rates in refractory glaucoma with a relatively favourable safety profile.

### What this study adds

- The IOP-lowering effect can be sustained beyond three years, demonstrating long-term efficacy.
- Nearly half of the patients required stent removal, leading to further IOP reduction without additional surgical intervention.
- Complications such as tube exposure and hypotony remain concerns but are manageable with appropriate interventions.
- This study may influence surgical decision-making, encouraging broader adoption of PGI with an intraluminal stent, and it supports future research on optimising stent timing and strategies to minimise complications.

## Data Availability

All datasets generated during and/or analysed during the current study are available from the corresponding author on reasonable request. The data that support the findings of this study are not publicly available because they contain informaOon that could compromise the privacy of research participants, but are available from CL upon reasonable request.
